# Endobolome, a New Concept for Determining the Influence of Microbiota Disrupting Chemicals (MDC) in Relation to Specific Endocrine Pathogenesis

**DOI:** 10.3389/fmicb.2020.578007

**Published:** 2020-11-30

**Authors:** Margarita Aguilera, Yolanda Gálvez-Ontiveros, Ana Rivas

**Affiliations:** ^1^Department of Microbiology, Faculty of Pharmacy, University of Granada, Granada, Spain; ^2^Instituto de Investigación Biosanitaria ibs.GRANADA, Granada, Spain; ^3^Department of Nutrition and Food Science, Faculty of Pharmacy, University of Granada, Granada, Spain

**Keywords:** microbiota, endocrine disrupting chemicals, endobolome, hormones, endocrine pathogenesis, microbiota disrupting chemicals

## Abstract

Endogenous steroid hormones and Endocrine Disrupting Chemicals (EDC) interact with gut microbiota through different pathways. We suggest the use of the term “endobolome” when referring to the group of gut microbiota genes and pathways involved in the metabolism of steroid hormones and EDC. States of dysbiosis and reduced diversity of the gut microbiota may impact and modify the endobolome resulting at long-term in the development of certain pathophysiological conditions. The endobolome might play a central role in the gut microbiota as seen by the amount of potentially endobolome-mediated diseases and thereby it can be considered an useful diagnostic tool and therapeutic target for future functional research strategies that envisage the use of next generation of probiotics. In addition, we propose that EDC and other xenobiotics that alter the gut microbial composition and its metabolic capacities should be categorized into a subgroup termed “microbiota disrupting chemicals” (MDC). This will help to distinguish the role of contaminants from other microbiota natural modifiers such as those contained or released from diet, environment, physical activity and stress. These MDC might have the ability to promote specific changes in the microbiota that can ultimately result in common intestinal and chronic or long-term systemic diseases in the host. The risk of developing certain disorders associated with gut microbiota changes should be established by determining both the effects of the MDC on gut microbiota and the impact of microbiota changes on chemicals metabolism and host susceptibility. In any case, further animal controlled experiments, clinical trials and large epidemiological studies are required in order to establish the concatenated impact of the MDC-microbiota-host health axis.

## Introduction

The effects of the bidirectional between steroid hormones and gut microbiota on the development of diseases have been recently reported (Kwa et al., 2016; Bajer et al., 2017). Hormones can have an impact on the composition and metabolism of the microbiota. In turn, the gut microbiome is highly involved in hormone homeostasis through a number of possible mechanisms. The concept “microgenderome” refers to the role of sex differences in gut microbiota in relation to the incidence and comorbidity of certain diseases (Mulak et al., 2014). An interesting example of gut microbiome/hormones interactions is the role of sex in type 1 diabetes found in a non-obese diabetic mouse model that revealed that exposure of female mice to androgens protected them from the disease (Fox, 1992; Markle et al., 2013), which suggests the role played by sex-specific interactions in disease development. The way this modulation affects hormone metabolism, physiology, and pathophysiology is still unclear. The gut microbiome can also modify hormone levels in the host by participating in hormone biotransformation. A key triad of sex hormones, host genotypic and phenotypic responses, and gut microbiome has been proposed (Rosenfeld, 2017).

Previous research works have dealt with the effects of diet, exercise and antibiotic use on host gut microbiome (Munyaka et al., 2014; Murphy et al., 2015; Xu and Knight, 2015; Zhang and Yang, 2016; Singh et al., 2017), but the effects of exposure to exogenous chemicals on the microbiota have been poorly investigated (Hu et al., 2016). Among these environmental contaminants, the Endocrine Disrupting Chemicals (EDC) have been recently defined by the Endocrine Society as: “an exogenous [non-natural] chemical, or mixture of chemicals, that interferes with any aspect of hormone action” (Zoeller et al., 2016). This interference with the endocrine system may result in biological, physical and metabolic disturbances in humans. EDC can bioaccumulate up the food chain and in the environment and that have been detected in humans worldwide. These chemicals include dioxins, pesticides, pyrethroids, polychlorinated biphenyls (PCBs), flame retardants and, antibacterial such as triclosan (García-Mayor et al., 2012; Darbre, 2017; Galvez-Ontiveros et al., 2020), plant derived compounds found such as phytoestrogens and plasticizers such as phthalates and bisphenol A (BPA). Exposure to EDC might result in marked disturbances in the gut microbiome, which in turn lead to widespread disruptions in several host systems and damage of the commensal bacteria in the host gut (Rosenfeld, 2017). However, the association between misbalanced microbiota diversity or dysbiosis and possible biological mechanisms responsible for the development of certain diseases in different environmental exposure settings remains largely unknown (Hu et al., 2016).

The term “estrobolome” was previously used to describe the gut microbiota genes involved in the synthesis of estrogen-metabolizing enzymes (Plottel and Blaser, 2011). We now suggest the use of the term “endobolome” when referring to the group of gut microbiota genes and pathways involved not only in the synthesis of estrogens, but also in the metabolism of other steroid hormones and endocrine disruptor chemicals. States of dysbiosis and reduced diversity of the gut microbiota may impact and modify the endobolome resulting at long-term in the development of certain pathophysiological conditions. The reduction in the diversity of microbial communities’ composition and inflammation could decrease the enzymatic activity which, sequentially, decreases the metabolization of hormones and EDC into their modified, active or inactive and circulating metabolites. This reduced availability of circulating hormones and EDC results in changes in hormone receptor activation, which may lead to the development of disorders related to demonstrated hormone deficiencies such as obesity, metabolic syndrome, and cardiovascular and related cognitive dysfunctions. Conversely, endobolome can also stimulate the production of gut microbial enzymes which leads to an increase in circulating hormones which, in turn, increase the risk of diseases related with high serum levels of hormones such as sex-hormone driven cancers.

The relevance of the crosstalk between EDC, hormones and gut microbiota warrants a review of the current information available on the interactions of these chemical disruptors with sex steroid hormones and gut microbiota as well on the role played by these interactions in the development of hormone-related diseases.


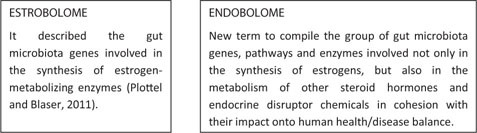


## Natural Hormone-Gut Microbiome Axis

Sex-related differences in gut microbome have been found in human and mice experimental models. These differences show a greater alpha diversity in the gut microbiome of women (Falony et al., 2016), and lower abundance of *Clostridia*, *Methanobrevibacter*, and *Desulfovibrio* in men (Falony et al., 2016). As in humans, a larger alpha diversity was found in female mice and a higher relative abundance of *Bacteroidetes* in males (Yurkovetskiy et al., 2013; Kozik et al., 2017).

There is evidence supporting the idea that sex-dependent differences in gut microbiome composition are related to sex steroid hormones (Table 1). In this respect, some studies have shown that gut microbiome in women differs from that of men after puberty and that gonadectomy results in disturbances in the microbiome or dysbiosis. For instance, during infancy there were no differences in the microbiota between fraternal twins of the same or different sex. Moreover, in different- or same-sex fraternal twins no differences in gut microbiota were found during childhood, but after puberty significant structural differences in gut microbiome were found in different-sex fraternal twins in comparison with same-sex twins (Yatsunenko et al., 2012). Similar sex-dependent changes were observed in rodent models after puberty (Markle et al., 2013; Yurkovetskiy et al., 2013) and after gonadectomy with a reduction in alpha diversity in ovariectomized mice and rats (Moreno-Indias et al., 2016; Choi et al., 2017). Ovariectomized mice showed decreased *Bacteroidetes* and increased *Firmicutes* versus controls (Choi et al., 2017). In male mice reduced *Bacteroidetes* and *Ruminococcaceae* were observed after gonadectomy compared to controls (Harada et al., 2016; Org et al., 2016). Future investigations should examine acute versus long-term effects of gonadectomy (with or without hormone replacement) in rodents and in other species of mammals.

**TABLE 1:** Studies linking natural hormones, the related diseases and the effects on the microbiota.

**References**	**Animal model**	**Microbiota location**	**Dysbiosis/Related condition**	**Microbiota changes**
**Studies in animals**				
Yuan et al. (2018)	C57BL6 mice	Gut	• Endometriosis induced the dysbiosis.	• Increased *Firmicutes/Bacteroidetes* ratio. • Increased *Bifidobacterium* (phylum *Actionobacteria*).
Bailey and Coe (2002)	Female rhesus monkeys	Gut	• Endometriosis is associated with an altered profile of intestinal microbiota.	• Decreased Lactobacilli. • Increased Gram-negative aerobic and facultative anaerobic bacteria.
Markle et al. (2013)	Diabetic and non-obese diabetic mice	Gut	• Childhood T1D may induce dysbiosis.	• Commensal colonization resulted in increased serum levels of testosterone and protected non-obese diabetic males from T1D. • In young females, the transfer of gut microbiota from adult males resulted in microbiota changes, with increased testosterone and metabolic changes, decreased inflammation of pancreatic islets and autoantibody production, and protection against T1D.
Guo et al. (2016)	Female Sprague-Dawley rats with PCOS	Gut	• PCOS may significantly alter the gut microbiome.	• Decreased *Lactobacillus, Ruminococcus*, and *Clostridium* and increased *Prevotella* were found in PCOS females. For *Bifidobacterium, Escherichia coli, Enterococcus*, and *Bacteroides* there was no significant differences between PCOS females and the control group. • Gut dysbiosis was related to sex hormone levels, estrous cycles and morphological changes in the ovaries.
Kelley et al. (2016)	Female C57BL/6N mice with PCOS	Gut	• PCOS may significantly alter the gut microbiome.	• A significant decrease in the overall species composition and phylogenetic diversity of the gut microbiota, particularly in the relative abundance of *Bacteroidetes* and *Firmicutes*, was observed.
**Studies in humans**				
Banerjee et al. (2017)	Women	Tumor ovarian tissue and non-tumor ovarian tissue	• Altered microbiome in ovarian tumor tissue.	• Increased *Proteobacteria* and *Firmicutes*. • Decreased *Bacteroidetes, Actinobacteria, Chlamydiae, Fusobacteria, Spirochaetes* and *Tenericutes.* • *Retroviridae, Hepadnaviridae, Papillomaviridae, Flaviviridae, Polyomaviridae* and *Herpesviridae* abound in > 50% of cancer samples. • *Pneumocystis*, *Acremonium, Cladophialophora*, *Malassezia*, and *Microsporidia Pleistophora* were significantly detected in all the ovarian cancer samples screened//in 100% of tumor samples examined.
Sfanos et al. (2018)	Men	Gut	• Prostate cancer hormonal therapy may alter the gut microbiota.	• Decreased alpha diversity in the gut microbiota of prostate cancer patients. • A significant increase in *Akkermansia muciniphila*, *Ruminococcaceae* spp., and *Lachnospiraceae* spp., was found in the fecal samples of prostate cancer patients undergoing oral ATT. Additionally, a significant decrease in the number of sequencing reads belonging to families such as *Brevibacteriaceae*, *Erysipelotrichaceae*, and *Streptococcaceae* was also observed.
Golombos et al. (2018)	Men	Gut	• An alteration of gut microbiota was observed in men with prostate cancer.	• Increased *Bacteriodes massiliensis* in prostate cancer patients versus controls. • Increased *Faecalibacterium prausnitzii* and *Eubacterium rectale* in controls versus prostate cancer patients.
Liu et al. (2017)	Women	Gut	• Disturbances in the gut microbiota of both obese and non-obese women with PCOS compared to non-obese controls.	• Decreased bacterial alpha diversity, increased LPS-producing bacteria, and decreased spore-forming bacteria species. • Increased *Bacteroides* and *Escherichia/Shigella* and decreased *Akkermensia* in obese women with PCOS.
Fuhrman et al. (2014)	Women	Gut	• Higher gut microbiota diversity was found in women with a high hydroxylated estrogens metabolites/parental estrogens ratio in urine.	• The relative abundances of the *Clostridia* class, including the *Clostridiales* order and the *Ruminococcaceae* family, were directly associated with the ratio of metabolites to parental estrogens, while the *Bacteroides* genus was inversely associated with this ratio.

*T1D: type 1 diabetes; PCOS: polycystic ovary syndrome; ATT: axis-targeted therapies.*

Microbiota, environmental and pathogenic bacteria have the capacity to metabolize steroid hormones and their related metabolites (Garcia-Gomez et al., 2013). Adlercreutz et al. (1984) showed that disturbances in the gut microbiota secondary to antibiotic therapy resulted in an increase in fecal estrogens in women, which suggests the involvement of microbiota in estrogen levels. Lombardi et al. (1978) demonstrated that fecal microbiota may metabolize parent estrogens and their metabolites. Fuhrman et al. (2014) demonstrated that high estrogen metabolites/parent estrogens ratios related to increased microbiota diversity in healthy postmenopausal women. In a mouse model, Markle et al. (2013) showed that early-life changes in gut microbiome result in increased testosterone and metabolic changes that can suppress autoimmune diseases in genetically high-risk animals while preserving fertility.

Gut microbiota can affect circulating levels of hormone metabolites by deconjugation of the conjugated forms secreted in bile and they can be subsequently reabsorbed through the mucosal wall. Fuhrman et al. (2014) showed that estriol, a hydroxylated estradiol metabolite, enters the enterohepatic circulation, and that urinary and fecal levels of estriol and parent estrogens are altered after antibiotic administration. They also suggest that gut microbiota may differentially affect total hormone levels as the association between total hormone and microbiota diversity, which seemed to be stronger for metabolites than for parent hormones. Lastly, they found higher gut microbiota diversity in women with higher levels of hydroxylated estrogen metabolites.

Shin et al. (2019) conducted a study in 31 men and 26 women and reported that sex steroid hormone levels are correlated with gut microbial diversity and composition. In men, they observed that testosterone levels were associated with increased *Acinetobacter*, *Dorea*, *Ruminococcus*, and *Megamonas*. In women, high estrogen levels were associated with increased *Bacteroidetes* and decreased *Firmicutes* phyla. Lastly, a strong association was found between *Slackia* and *Butyricimonas* and estradiol levels.

The essential role played by gut bacteria in hormone metabolism was described decades ago (Janssen and Kersten, 2015). Gut colonization by bacteria in germ-free mice resulted in the regularization of the estrous cycle in females and increased sperm counts in males, which in turn led to the repair of fertility and reproductive capacities (Shimizu et al., 1998). In a cross-sectional study conducted by Fuhrman et al. (2014) that included 60 healthy postmenopausal women, the authors examined the relationship between the composition and diversity of microbiome, assessed trough 16S rRNA gene sequencing, with measurements of urinary levels of estrogens and their metabolites. Authors found a significant correlation between microbiota diversity and the estrogen metabolites/parent estrogens ratio in urine. Moreover, this ratio increased with increased microbial phylogenetic diversity. These findings support the hypothesis that differences in estrogen levels and their metabolism significantly correlate with alterations in gut microbial diversity.

All these studies compiled above revealed significant evidence of hormonal regulation of the microbe-controlling mechanisms, supporting sex differences in microbiota, in addition to sex-specific responses to the same microbiota. The relationship between microbiota, hormones and metabolism seems multi-directional. Collectively, these studies indicate that upcoming investigations into the composition and function of the microbiota must carry on dividing data for sex-differential interactions to understand these complex connections more entirely.


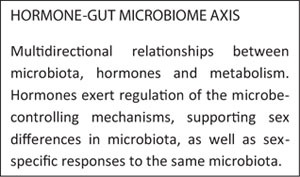


**FIGURE 1:**
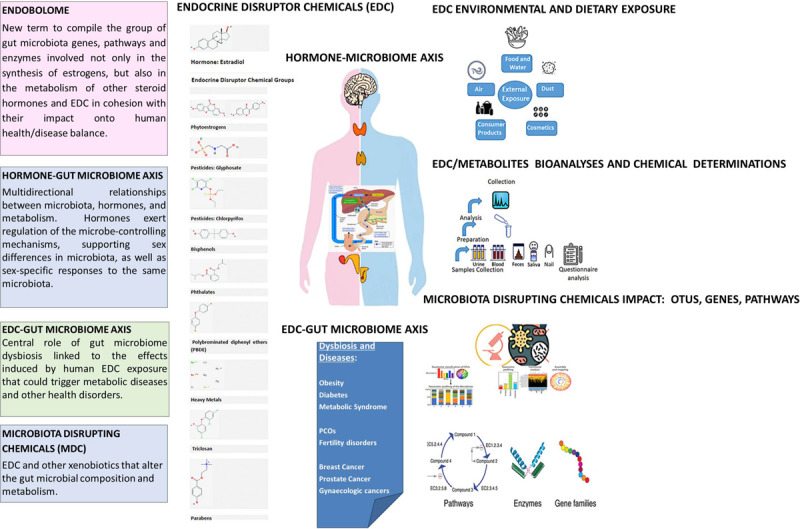
Endobolome: multiproxy approach to determine the influence of microbiota disrupting chemicals (MDC) in relation to specific endocrine pathogenesis.

The determination of the endobolome experimental parameters may serve as potential biomarkers of certain diseases (Figure 1). For example, the determination of β-glucuronidases may help in the identification of some specific bacterial species and, as mentioned above, this enzyme might potentially increase the intestinal reabsorption of endogenous hormones and EDC. Microbial β-glucuronidases will process endogenous molecules like β-glucuronides of hormones and exogenous β-glucuronides from the diet by enabling these compounds to bind to hormone receptors and subsequently produce their physiological effects (Baker et al., 2017). Phytoestrogens also exert their activity via mechanisms involving hormonal receptors. As mentioned above, increased estrogen metabolites correlate with increased microbiota diversity versus parent hormones in fecal samples (Fuhrman et al., 2014). Additionally, a higher parent hormones/hormone metabolites ratio correlates with an increased risk of breast cancer (Samavat and Kurzer, 2015), obesity and other metabolic diseases (Chen and Madak-Erdogan, 2016).

Interestingly, androgens also play a central role in sexual physiology, and disturbances in their action are associated with the development of different conditions in both men and women. Collden et al. (2019) demonstrated that glucuronidases produced by the cecal microbiota can degrade the high content of testosterone and dihydrotestosterone found in the small intestine. The high content of free or unconjugated dihydrotestosterone found in the colon of healthy young mice of both sexes and men suggests that gut microbiota might be involved in the metabolism of intestinal androgens and can therefore affect the risk of androgen-related diseases in the distal intestine and probably also in remote anatomical sites.

Identification of the bacterial genes in the gut microbiota that encode enzymes responsible for the biotransformation of endogenous steroids could explain potential interactions between gut microbiome, host and the development of certain diseases. *In vitro* studies have shown the capacity of gut microbiota to the biotransformation of steroids. In this respect, Ervin et al. (2019) conducted the first *in vitro* study to determine the capacity of 35 β-glucuronidases of human gut to reactivate estrone-3-glucuronide and estradiol-17-glucuronide to their parent compounds. This supports the idea that the estrobolome is a complex series of processes that occur in the gastrointestinal tract of mammals and the probably involves different enzymes, including distinct types of β-glucuronidases. Ridlon et al. (2013) showed that *Clostridium scindens* may transform primary bile acids to toxic secondary acids, and remove the two-carbon side chain of glucocorticoids transforming them into androgens. Doden et al. (2019) demonstrated that 20 β-hydroxysteroid dehydrogenase from *Bifidobacterium adolescentis* can reduce host glucocorticoids and thereby serve as potential probiotics for the management of androgen-related conditions.

Another possible mechanism of interaction between EDC, endogenous sex steroid hormones and gut microbiota is that dysbiosis caused by EDC and hormone imbalance levels may increase the permeability of the intestinal mucosa barrier in the host. This would allow pathogens, LPS, toxins and metabolites to enter the circulatory system thereby promoting chronic low-grade systemic inflammation in the host, even targeting organs like the brain (Fuhrman et al., 2014; Pickard et al., 2017).

Another potential mechanism involves the capacity of gut microbiota to metabolize EDC and host hormones to produce novel hormone receptor ligands (Schug et al., 2011). Additionally, the microbiota may be modified in order to generate lower-affinity hormones with endocrine functions but with a reduced risk of developing hormone-responsive diseases like breast cancer (Chen K. L. A. et al., 2018). It has been showed in a mouse model that the status of hormone receptors such as estrogen receptor β may alter microbiota composition and this microbiota behaves differently to changes from diet complexity, promoting Proteobacteria enrichment (Mulak et al., 2014). This suggests that sex hormones have the capacity to mediate and modulate growth, metabolism, and virulence of bacterial pathogens.

Bringing together all these findings allow us to validate the theory that the endobolome implies a multidimensional set of processes on-going inside the gastrointestinal tract that possible involves many enzymes and pathways. The capacity of microbes to control hormones and of hormones to modify microbial diversity must be considered in future studies. Furthermore, it is possible that in complex microbial communities the functions of hormone control could be diverse between different members of the community. The detection of crucial microbial enzymes in biodegradation might support to find microbial hormone degradation routes and biomarkers of hormones metabolism by a microbial community.

Moreover, we suggest that the gut may act a reservoir for hormone metabolites that could exert effect locally and possibly in the distance in systemic homeostasis and the development of disease. Hormones and microbiota could exert their activity separately for the development of disorders and diseases; however, recent studies have shown that hormones and microbiota may function together in conditions related to hormone hypersecretion or deficiency (Tetel et al., 2018). However, the way certain hormones alter the microbiome to confer normal metabolic phenotypes or how microbial hormone metabolites act as ligands binding to hormone receptors to mediate metabolic functions (Fukui et al., 2018).

The communication between gut microbiota and hormones guides to biological modifications through a diversity of tissues ranging from neuronal development to reproductive health. When dysibiosis occurs, these physiological responses are altered and contribute to development of disease. Polycystic ovary syndrome (PCOS) is a chronic metabolic disease in reproductive-aged women with prevalence between 3 and 26% and whose pathogenesis is currently unknown. A novel hypothesis suggests that microbiota dysbiosis may promote the production of androgens in the ovaries which results in disordered folliculogenesis triggered by a chronic inflammatory response and insulin resistance (Tremellen and Pearce, 2012). Kelley et al. (2016) found in a letrozole (a non-steroidal aromatase inhibitor) induced PCOS murine model that treatment with letrozole significantly changed the gut microbiome composition in a time-dependent manner with a reduction in overall species and phylogenetic richness. Guo et al. (2016) transplanted fecal microbiota from healthy rats to letrozole-induced PCOS mice and found that transplantation resulted in normalization of the estrous cycles and the ovarian morphology. These findings suggest the association between sex hormone levels, estrous cycles, ovarian morphology and gut microbiota composition. Another murine model study showed that transplantation of male cecal microbiota to females led to increased testosterone levels, compared with unmanipulated control females and female mice transplanted with female cecal microbiota (Markle et al., 2013). Liu et al. (2017) conducted a study in 33 PCOS patients and found that gut dysbiosis correlated with the disease. The authors also reported an increase in the relative abundance of *Bacteroides*, *Escherichia*/*Shigella*, and *Streptococcus* in PCOS individuals, a negative correlation between these bacterial species and ghrelin levels, and a positive correlation with testosterone and body mass index (BMI). Lastly, decreased abundances of *Akkermansia* and *Ruminococcus* in PCOS, with a negative correlation of these species and body-weight, sex-hormone, and brain–gut peptides. Summarizing, human microbiota seems to play a key role in endocrine and reproductive system disorders. Moreover, microbiota reproductive dysbiosis have started to be treated by probiotics using typical species from genus *Lactobacillus* (Lopez-Moreno and Aguilera, 2020). Some *Bifidobacteria* seems modulate sex hormone levels in patients with PCOS through the gut–brain axis (Zhang et al., 2019).

The prostate is an estrogen and androgen target tissue and estrogens have been shown to promote growth and differentiation of prostate, potentially leading to the development of prostate cancer. Recent studies indicate that gut microbial species leading to increased serum levels of estrogens may increase the risk of prostate cancer (Nelles et al., 2011). Androgens are involved in the growth and survival of normal prostate cells and in the development of prostate cancer, with androgen suppression therapy being a usual treatment for this type of cancer. However, although most patients with advanced metastatic prostate cancer respond to androgen deprivation, the tumors eventually become androgen-independent (Feldman and Feldman, 2001; Porter et al., 2018). The capacity of gut microbiome to alter androgen levels may result in depletion, less effective androgen suppression therapy. Sfanos et al. (2018) found a distinct microbial composition in patients undergoing oral androgen suppression therapy versus patients not treated with this therapy. Treated patients also showed an increase in the bacterial metabolic pathways that stimulate androgen synthesis. In addition, significant differences were found in the gut microbial composition between prostate cancer patients and patients with benign lesions (Golombos et al., 2018). Similarly, Magri et al. (2018) considered prostatitis as a systemic diseases correlating with alterations of specific microbiota or seminal dysbiosis.

The relation between the gut microbiome and the development of gynecologic cancers remains unclear (Mert et al., 2018), but there is growing evidence of the role played by changes in microbiome in an increased risk of these types of cancers. It is plausible that all these factors can lead to cancer by modifying the microbiota and its interaction with host DNA or just simply by triggering inflammatory reactions. Some possible mechanisms related to this increased risk are the capacity of the microbiota to interact with the host DNA and to promote chronic inflammation. As for epithelial ovarian cancer patients, Banerjee et al. (2017) found a distinct gut microbiota composition versus controls. *Retroviridae* and human papilloma virus as well as *Proteobacteria* and *Firmicutes* were found in ovarian cancer patients versus controls (Mitra et al., 2016). In agreement with this hypotheses, probiotic microorganisms are been investigating recently by their antigenotoxic potential effects (Janosch et al., 2019).

Breast cancer has also been related with disturbances in the microbiota. In this respect, it has been reported a decreased gut microbial diversity and a different composition in postmenopausal women with breast cancer versus controls (Zhu et al., 2018). Lahiji et al. (2020) analyzed recently the effect of symbiotic on glycemic profile and sex hormones in overweight and obese breast cancer survivors following a weight-loss diet through a randomized controlled trial. However, Jones et al. (2019) did not found any correlation between fecal microbiota and breast density on the mammograms.

As with gut microbiota, the enteroendocrine cells found in the gastrointestinal tract can alter the microbial composition having an effect on the intestinal health patterns of estrogen metabolism used as markers of breast cancer risk in postmenopausal women have been correlated with gut microbiota diversity and composition (Kwa et al., 2016).

Estrogens promote the growth and proliferation of epithelial cells in the mammary gland and in the reproductive system and are thereby associated with proliferative conditions like endometrial cancer and endometriosis. Several authors have suggested an association between gut microbiota alterations and development of endometriosis (Ata et al., 2019). Bailey and Coe (2002) supported this equivalent idea association in a rhesus monkey model. In addition, in a murine model Yuan et al. (2018) reported a distinct composition of gut microbiota 42 days after endometriosis induction.

Taking together all this data shown that commensal microbiota of the host could be implicated in gender bias existing in numerous diseases. The capacity of the gut microbiome in controlling hormone levels allow to have stimulating therapeutics applications in different diseases, taken into account a sex-tailored therapeutic approach.

Efficacy and safety of current hormone replacement therapies might be improved by combining probiotics with the hormones that work independently or synergistically to provide a more holistic treatment approach against hormonal disease (De Franciscis et al., 2019). Lastly, it has been suggested that probiotic bacteria administered in combination with hormone replacement therapy may increase the efficacy of hormone replacement and attenuate the side effects that can arise from its use, therefore providing a more comprehensive treatment of hormonal diseases. Next generation probiotics will explore the function in this area of knowledge (Ejtahed and Hasani-Ranjbar, 2019). The response to therapeutic alteration of the microbiota using prebiotics, live biotherapeutics or fecal transplant therapy is likewise probable to be dissimilar in males and females, although this has not been specifically studied to date.

The studies compiled revealed significant evidence of sex-specific communities, the role of sexual maturation producing changes to microbial communities, and proof that microbial communities could have a key function by modulating hormonal and metabolic pathways. Results highlight the need to examine sex-specificity in microbial composition and function.

## Endocrine Disruptors-Gut Microbiome Axis

The role played by human exposure to EDC in the development of diseases such as breast cancer, diabetes, obesity and some neurobehavioral disorders is widely known (Andujar et al., 2019). These diseases have also been related to gut dysbiosis, which suggests the central role of gut microbiome in the effects induced by EDC exposure in human metabolism (Galvez-Ontiveros et al., 2020). It can therefore be suggested that the exposure to EDC may result in alterations in the gut microbiome which may ultimately affect human health. In a bidirectional interaction, microbiota can metabolize EDC to biologically active or inactive forms, and EDC may induce the proliferation and growth of certain bacteria. These EDC-induced changes in the gut microbiota may lead to disturbances in different host systems. However, it remains unclear if EDC-induced metabolic disruptions in the host occur before the changes in the microbiome or if the EDC-induced microbiome changes result in metabolic disruptions (Rosenfeld, 2017).

The gastrointestinal tract is the main route of entry for EDC; however, their absorption through the intestinal wall is low and they are transported by the peristaltic movement to the distal small intestine and caecum where microbial flora is more abundant and they can be directly metabolized by the microbiota thereby increasing or decreasing their toxicity to the host. Part of the disruptors is taken to the liver by the portal circulation where they are conjugated. These conjugated forms can be excreted in the bile entering the small intestine where microbiota tend to deconjugate them therefore restoring the original compound or producing new toxic metabolites. EDC can also affect the composition and metabolic activity of gut microbiota, which in turn may result in a disrupted activity of EDC metabolites or in the toxicity of other contaminants metabolized by the gut microbiota (Claus et al., 2017). Data from animal models suggest that the alterations in gut microbiota not only affect the levels of microbial enzymes, but also the levels of hepatic enzymes in the host (Snedeker and Hay, 2012). In addition, exposure to EDC could alter gut microbiota functions.

The gut microbiota has the capacity to transform non-EDC in active compounds. For example, Van de Wiele et al. (2005) reported that colonic microbiota can metabolize polyaromatic hydrocarbons into 1-hydroxy pyrene and 7-hydroxybenzo[a]pyrene, biologically active estrogen metabolites, while the gastrointestinal digestion of these xenobiotics did not produce estrogen metabolites. This finding suggests that the colonic microbial communities have the capacity to transform parent compounds directly into active metabolites.

The hypothesis that exposure to EDC may result in alterations of the gut microbial composition has not yet been extensively explored. In this scenario, the microbiota and their products may act as mediators of the effects caused by these contaminants, which will eventually lead to the development of disorders and diseases. Several EDC have been shown to promote dysbiosis or to inhibit bacterial growth in *in vitro* and in *in vivo* models (Galvez-Ontiveros et al., 2020). Dysbiosis has been associated with intestinal and non-intestinal disorders which have been in turn related to EDC exposure. This would be consistent with the idea that EDC exposure may have an impact on the normal gut microbiota colonization, which will ultimately affect host physiology and health. These EDC-induced alterations of the gut microbiota composition may represent an underestimated mechanism of interfering with human health.

## Microbiota Disrupting Chemicals (MDC)

We propose that EDC and other xenobiotics that alter the gut microbial composition and metabolism should be categorized into a subgroup termed “microbiota disrupting chemicals” (MDC). This will help to distinguish the role of contaminants from other microbiota disruptors such as diet, environment, physical activity and stress. These MDC might have the ability to promote changes in the microbiota that can ultimately result in intestinal, hormonal and chronic or long-term systemic diseases in the host as illustrated in Figure 1.

Below we summarize the best known and studied by now MDC and their effects on the microbiota.


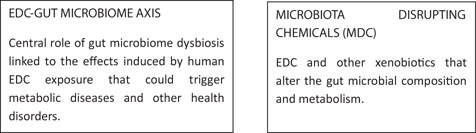


### Pesticides

Several studies have revealed the central role played by gut microbiota in the toxicity of pesticides, with important implications for environmental, animal and human health (Yuan et al., 2019; Table 2). Glyphosate and chlorpyrifos, the most widely used herbicide and pesticide, respectively, exhibit endocrine disrupting activity. Human exposure occurs mainly through diet and drinking water (Gillezeau et al., 2019). They have been reported to interfere with gut microbial communities and enteroendocrine cells (Li et al., 2019). Mucosal-associated invariant T-cells (MAIT) are activated by bacterial-derived metabolites from riboflavin and folic acid biosynthesis. Mendler et al. (2020) investigated the effects of chlorpyrifos and glyphosate on the regulation of MAIT activity but gut microbiota and found that exposure to these pesticides results in disturbances in the gut microbial communities studied, thereby promoting a pro-inflammatory immune response.

**TABLE 2:** Studies linking exposure to pesticides and gut microbiota changes.

**References**	**Animal model**	**Pesticide dose**	**Microbiota changes**	**Health effects**
***In vitro* studies**				
Mendler et al. (2020)	Humans	CPF (50–200μM) or GLP (75–300 mg/L)	• The growth of *E. coli, Bifidobacterium adolescentis* and *Lactobacillus reuteri* was not inhibited.	• *E. coli* activated a significantly higher number of MAIT cells to produce TNFα and IFNγ after exposure to CPF. However, *B. adolescentis* and *L. reuteri* did not induce activation of MAIT cells *per se* after exposure to CPF or GLP. • *E. coli* decreased riboflavin production after exposure to CPF. In contrast, folate production by *E. coli* was significantly increased after exposure to CPF. • GLP exposure resulted in MAIT cell activation by *E. coli*, but exposure did not affect vitamin production. • Exposure to CPF and, to a lesser extent, GLP, can result in bacterial metabolism changes, with imbalances in the levels of activation/inhibition of bacterial metabolites that could have an impact on the inflammatory immune responses.
**Studies in animals**				
Wang et al. (2019)	Zebrafish	CPF (30, 100, and 300 μg/L)	• Significant decrease in *γ-Proteobacteria*.	• CPF induces oxidative stress. • Exposure to CPF could induce liver disorders of glucose and lipid metabolism in adult zebrafish.
Liang et al. (2019)	C57Bl/6 and CD-1 mice	CPF (5 mg/kg)	• Decreased *Bacteroidetes* phyla. • Increased *Proteobacteria* phyla.	• Exposure to CPF resulted in disrupted integrity of the intestinal barrier, which increases the entry of lipopolysaccharides into the body and lastly low-grade inflammation. • Increased fat mass. • Decreased insulin sensitivity.
Tang et al. (2020)	Male Sprague Dawley rats	GLP (0, 5, 50, and 500 mg/kg)	• Decreased *Firmicutes* phylum and *Lactobacillus* genus. • Increased pathogenic bacteria *Clostridium botulinum*.	• GLP exposure decreased the villus height/crypt depth ratio in the duodenum and jejunum which is associated to decreased digestive and absorptive capacity. • Decreased activity of antioxidant enzymes • GLP exposure promotes the production of proinflammatory factors (IL-1β, IL-6, TNF-α, MAPK3, NF-κB and Caspase-3).
Li et al. (2019)	Male Wista**r** rats	CPF (0.3 mg/kg)	• Increased relative abundances of *Streptococcus*, *Ruminiclostridium*, and *norank_f_Coriobacteriaceae* in adult rats exposed to CPF. • Reduced relative abundances of *Corynebacterium_1, Psychrobacter, Facklamia, norank_f__Peptococcaceae, Oligella, Brevibacterium, Catabacter, Dietzia, Atopostipe*s and *Ignavigranum* in newly weaned rats exposed to CPF.	• Decreased concentrations of luteinizing hormone, follicle-stimulating hormone, and testosterone were found in rats exposed to CPF and fed normal-fat diet. The counteracted effect of the high-fat diet was also found in intestinal hormones and pro-inflammatory cytokines. • TNF-α were found in newly weaned rats exposed to CPF, whereas only peptide YY, ghrelin and IL-6 increased significantly in rats exposed in adulthood.
Gao et al. (2017a)	C57BL/6 mice	Diazinon (4 mg/L)	• Decrease in several genera of the *Lachnospiraceae* family in exposed male and female mice. • High prevalence of pathogenic bacteria (*Burkholderiales* and *Erysipelotrichaceae_Coprobacillus*) was only found in exposed male mice. As well as increased *Bacteroidetes* phylum and decreased *Firmicutes*. • *Lachnospiraceae, Ruminococcaceae, Clostridiaceae* and *Erysipelotrichaceae* decreased in female mice.	• Tryptophanase was significantly downregulated in male mice but not in females. • Glycine was decreased in male mice, but not in females. • Exposure to diazinon resulted in disturbances of bile acid metabolism in both male and female mice, but the patterns and involved metabolites were sex dependent. Lithocholic acid and cholesterol increased in males. Lithocholic acid also increased in females, but with decreased cholesterol.
Gao et al. (2017b)	C57BL/6 mice	Diazinon (4 mg/L)	• Intestinal dysbiosis was observed in the previous study (Gao et al., 2017a).	• Altered quorum sensing mechanisms. • Increased motility and genes related to sporulation. • Activation of stress response pathways. • Impaired metabolic homeostasis of carbohydrates, fatty acids, and amino acids.

*CPF, chlorpyrifos; GLP, glyphosate; MAIT cell, Mucosal-associated invariant T-Cell; TNFα, tumor necrosis factor-α; IFNγ, interferon-γ.*

In a fish model, chlorpyrifos led to disorders in the hepatic lipid metabolism linked to oxidative stress in the gut and dysbiosis (Wang et al., 2019). In a murine model, Liang et al. observed that chlorpyrifos induced changes in the gut microbiota which resulted in an increased risk of obesity and insulin resistance (Liang et al., 2019). They also found that this pesticide can disrupt the integrity of the intestinal barrier, leading to the entry of lipopolysaccharides into the bloodstream ultimately resulting in low-grade inflammation.

Using 16 S rRNA gene sequencing in a rat model, Tang et al. (2020) observed that glyphosate exposure led to a significant increase in α-diversity and changes in gut microbiota composition with decreased relative abundance of Firmicutes and Lactobacillus and enrichment of potentially bacterial pathogens. *Firmicutes* produces butyrate as a byproduct of fermentation. It has been reported that butyrate-producing bacteria play a role in intestinal epithelial barrier maintenance (Canani et al., 2011). Rueda-Ruzafa et al. (2019) showed that glyphosate-mediated inactivation of 5-enolpyruvylshikimate-3-phosphate synthase from intestinal microbiota results in dysbiosis which in turn led to alterations in central nervous system causing emotional, neurological and neurodegenerative disorders.

In a rat model, Li et al. (2019) investigated the effects of chronic exposure to chlorpyrifos on serum hormone levels, pro-inflammatory cytokines and gut microbiota and found that exposure significantly decreased levels of luteinizing hormone, follicle stimulating hormone and testosterone and change gut microbiota composition. The affected bacteria included short-chain fatty acid-producers, which stimulate the hypothalamic-pituitary-adrenal axis and the immune response, and increase the permeability of the gut barrier.

Diazinon, an organophosphate pesticide with estrogenic activity (Manabe et al., 2006) has shown to alter the structure of the gut microbiome community, functional metagenome, and their associated metabolic profiles in a sex-related manner in murine models (Gao et al., 2017a). Gao et al. (2017a) suggest that diazinon neurotoxicity and its sex-dependent effects are related to the pesticide-induced alterations of the gut microbiome and its functions. The same authors (Gao et al., 2017b) investigated the effects of diazinon exposure by determination the gut bacterial metatranscriptome. They demonstrated that diazinon has the capacity to regulate quorum sensing, a system used to regulate bacterial population density and composition, and more importantly, their functional genes. Lastly, they also found that diazinon exposure results in the activation of stress response pathways and in disruptions of energy metabolism of gut bacteria.

Organochlorine pesticides with endocrine disrupting capacity have been also related with alterations in gut microbiota. Dichlorodiphenyldichloroethylene (DDE) exposure of 3-week-old male Sprague Dawley rats led to an increase of fat and body weight, dysregulation of glucose homeostasis, and promoted dysbiosis as suggested by a higher *Firmicutes/Bacteroidetes* ratio, resulting in disruptions in energy harvesting. DDE also resulted in alterations in the lipid metabolome profile with high plasma concentrations of phosphatidylcholine, phosphatidylserine, hosphatidylethanolamine, and triacylglycerol. The presence of these lipid metabolites was significantly associated with changes in microbiota composition (Liang et al., 2008).

### Bisphenol and Phthalates

Bisphenol A (BPA) is an environmental contaminant with endocrine disrupting activity widely used in the manufacture of polycarbonate plastics and epoxy resins and is released in large volumes into the environment. Phthalates are used in many household products and in food packaging acting as binding products and plasticizers. Human exposure to these contaminants occurs mainly through the diet, as they leech from plastic packaging, water, dust and to lesser extent from skin contact (Larsson et al., 2017). Both endocrine disrupters have shown to affect gut microbiota composition and function in animal models (Table 3). In a mouse model, DeLuca et al. (2018) found that BPA has the capacity to promote colonic inflammation and alter the levels of tryptophan and other microbiota metabolites derived from aromatic amino acids related to autoimmune diseases, specifically to inflammatory bowel conditions. In a mouse model, Lai et al. (2016) used 16S rRNA gene sequencing of cecal microbiota to demonstrate that BPA exposure through diet may result in alterations of gut microbiota composition and function, promoting the growth of *Proteobacteria*, a marker of dysbiosis, that has been associated to different disorders such as metabolic syndrome and inflammatory bowel disease (Zhang et al., 2006). Increased *Helicobacteraceae* and decreased *Firmicutes* and *Clostridia* populations were observed in BPA-fed mice. In a mouse model, Javurek et al. (2016) used 16s rRNA sequencing to show that BPA exposure, especially during development, results in generational and sex-related changes in the gut microbiome. Exposure to BPA induced in the parents and their offspring the growth of bacteria related to an increased risk of inflammatory bowel disease (IBD), metabolic disorders, and colorectal cancer. Also, in a murine model, Malaise et al. (2017) observed that perinatal exposure to BPA promoted disturbances in gut microbiota and immune system through a decrease in the Th1/Th17 cell frequencies in the lamina propria as well as an increase of Th1 andTh17 responses in the spleen. These early-life alterations are associated with impaired glucose sensitivity and IgA secretion into feces, and a drop of fecal *bifidobacteria* versus non-exposed mice.

**TABLE 3:** Studies linking exposure to bisphenol and phthalate and gut microbiota changes.

**References**	**Animal model**	**BPA or phtalates dose**	**Microbiota changes**	**Health effects**
***In vitro* studies**				
Lei et al. (2019)	C57BL/6J mice	DEHP (10 or 100μM)	• Increased abundance of *Lactobacillus* and *Parabacteroides*. • Decreased abundance of *Fluviicola* and *Enterococcus*.	• Exposed cultured microbiota increased the production of metabolites typically associated with fermentation of sugar and amino acid residues. • Among the metabolites detected are potentially toxic compounds derived from aromatic amino acid. Metabolites normally found in low concentrations in the intestine of healthy individuals but in high concentrations in developmental disorders were also detected [3-phenylpropionic acid and 3- (3-hydroxyphenyl) propionic acid are precursors of 3- (3 -hydroxyphenyl) -3 hydroxypropionic].
**Studies in animals**				
DeLuca et al. (2018)	C57BL/6 mice	BPA (50 μg/kg)	**_**	• Exacerbation of colon inflammation in animals with dextran sulfate sodium-induced colitis. • Reduction of fecal tryptophan and aromatic amino acids.
Lai et al. (2016)	CD-1 mice	BPA (BPA content in contaminated diet)	• Increased phylum *Proteobacteria* and Helicobacteraceae family. • Decreased *Firmicutes* and *Clostridia.*	• Absorption of BPA in the diet affects gut microbial composition, which relates with increased risk of metabolic disorders and inflammatory bowel disease.
Javurek et al. (2016)	California mice	BPA (50 mg/kg)	• Exposure to BPA in P0 females resulted in increased Mogibacteriaceae, *Sutterella* spp. and Clostridiales. BPA exposure of F1 females resulted in increased *Bifidobacterium* and the family Mogibacteriaceae. • In P0 males exposure led to increased abundance of *Mollicutes* and *Prevotellaceae* and increased. *Akkermansia* and *Methanobrevibacter* in F1 males	• BPA binds to and activates the estrogen receptors. • Many bacterial genera associated to EDC exposure are also found in several diseases such as inflammatory bowel disease, metabolic disorders, and colorectal cancer.
Malaise et al. (2017)	Mice	BPA (50 μg/kg)	• Decreased bifidobacteria and *Clostridium. butyricum* and *Clostridium Cluster XIVa*.	• Obesogenic impact of early and intrauterine exposure to BPA. • Glucose intolerance and decreased insulin sensitivity were observed in young and adult mice perinatally exposed to BPA. • Perinatal BPA exposure may also result in a loss of intestinal barrier.
Chen et al. (2018a)	Zefrafish	BPA (0, 2, and 20 μg/L)	• BPA exposure in male fish resulted in increased abundance of *Actinobacteria* at 13.9% (P < 0.001), *Hyphomicrobium*, and *Lawsonia*. • Single BPA exposure in female fish resulted in increased abundance of *Actinobacteria* to 15.7% (*P* < 0.001) and *Hyphomicrobium*.	• Exposure to 2 μg/L BPA resulted in decreased body weight in male fish versus controls, also in a significant reduction of the condition factor, and decreased intestinal levels of serotonin. • No significant differences in body length, body weight, or condition factor were observed in female fish. Single exposure to BPA resulted in significantly higher intestinal levels of serotonin versus controls. • Decreased levels of intestinal IL1β were observed in males versus significantly higher levels in females.
Xu et al. (2019)	Mice	BPA (30 or 300 μg/kg)	• Exposure to low BPA dose resulted in increased *Bacteroidetes* in non-obese diabetic female mice. Exposure to high dose increased unclassified bacteria from *Rikenellacaea* and decreased unclassified bacteria from *Bacteroidales* and *Lactobacillus*. • Sub-acute exposure in males resulted in an increase in bacteria classified as other at the phylum level was significantly increased from the low dose, while *Tenericutes* was significantly decreased in both BPA doses of exposure. At the genus level, exposure to high BPA dose increased *Odoribacter* and unclassified *Campylobacterales* and decreased *Anaeroplasma* and *Camplybacter*, while *Lactobacillus* was decreased at low BPA dose.	• BPA accelerated the development of T1D in female mice, but delayed the development of the disease in males. • The gut microbiome profile was consistently pro-inflammatory in females, but males had an overall decrease in anti-inflammatory and pro-inflammatory gut microbes.
Lei et al. (2019)	C57BL/6J mice	DEHP (1 or 10 mg/kg)	• Increased *Lachnoclostridum* abundance. • Decreased *Akkermansia, Odoribacter* and *Clostridium sensu stricto.*	• Diethylhexyl phthalate promoted p-cresol production but inhibited butyrate synthesis. • Exposure to diethylhexyl phthalate could be involved in neurodevelopmental disorders associated with dysbiosis.
Adamovsky et al. (2020)	Zebrafish	DEHP (3mg/kg)	• Diethylhexyl phthalate exposure increased *Bacteroidiales* and *Gammaproteobacteria* and decreased *Verrucomicrobiae* in males and females. • In males the abundance of *Fusobacteria* and *Betaproteobacteria* increased and *Saccharibacteria* decreased.	• Exposure in males negatively affected several membrane transport proteins, organic anion transporting polypeptides encoded by *slc* genes. • Altered metabolites detected by intestinal and immune Th cells.
Wang et al. (2020)	Sprague-Dawley and Wistar rats, BALB/c and C57BL/6J mice.	DEHP (0, 300, 1,000, and 3,000 mg/kg)	• Sprague-Dawley rats showed an increase in the Firmicutes/Bacteroidetes ratio and in the abundance of *Proteobacteria.* • In C57LB/6J mice Proteobacteria and *Actinobacteria* showed a downward trend. *Tenericutes* showed only a significant increase in cecal content. At the genus level, a decreased abundance of *Prevotella, Lachnospiraceae*, and *Desulfovibrio* was found. • In BALB/c mice only *Bacteroides* decreased, while Runimococcaceae and Rikenellaceae showed a significant increase. • Wistar rats showed increased abundance of *Adlercreutzia*, Eubacateriaceae and *Roseburia*, and a decrease of *Coprococcus*, Dehalobacteiaceae.	• Sprague-Dawley rats were more sensitive to exposure to DEHP with more severe organic damage, the highest Th1 inflammatory response and highest increase in body weight. Liver index increased in the medium and high dose groups. IL-2, IFN-γ, and TNF-α increased significantly and testosterone decreased. • In mice, only C57LB/6J mice exposed to high dose showed a higher liver index. Alanine aminotransferase, aspartate aminotransferase, alkalinephosphatase levels increased markedly in the highest dose group. • In Wistar rats, exposure to DEHP induced only a significant increase of alanineaminotransferase in the low and medium dose groups. • In BALB/c mice testosterone concentration decreased.
**Studies in humans**				
Yang et al. (2019)	Newborns	DEHP exposure in through medical treatment	• Firmicutes/Bacteroidetes ratios changed significantly. • *Bifidobacterium longum, Rothia* spp. and *Veillonella* decreased.	• *Rothia* and *Veillonella* abundance has been associated with a lower incidence of asthma. • Early exposure to diethylhexyl phthalate can alter the immune response in adulthood.

*BPA, bisphenol A; DEHP, Diethylhexylphthalate; TNF-α, tumor necrosis factor-α; IFNγ, interferon-γ, T1D, type 1 diabetes.*

Chen et al. (2018a) found that simultaneous exposure to titanium dioxide nanoparticles and BPA in zebrafish resulted in growth and gut microbiota alterations including loss of the mucosal barrier integrity and increased inflammation and oxidative stress. In addition, the changes in gut microbiota were sex- and concentration-dependent. The authors also found a correlation between zebrafish body weight and abundances of *Bacteroides*, which was also closely associated with the genera *Anaerococcus*, *Finegoldia*, and *Peptoniphilus*. They also reported that BPA at low concentrations interacts antagonistically with titanium dioxide but BPA at higher concentrations interacts synergistically. Lai et al. (2016) found that dietary BPA exposure in mice resulted in changes in gut microbiota structure similar to those found in mice on high-fat and high-sucrose diets. Xu et al. (2019) described that the effects of BPA-exposure on type 1 diabetes (T1D) in non-obese diabetic mice and found that were sex- age-dependent with changes in gut microbiota and inflammation being the main mechanisms disease worsening in juvenile exposure, while decreased inflammation resulted in attenuated T1D in perinatally exposed females.

In a mouse model Lei et al. (2019) showed that exposure to diethylhexylphthalate (DEP), a chemical associated with neurodevelopmental disorders, led to changes in gut microbial structure and metabolite profile. These changes in the microbiota may activate the production of *p*-cresol, a potentially toxic metabolite that has also been correlated with neurodevelopmental disorders. Recently, Adamovsky et al. (2020) showed that DEP exposure results in alterations in the microbiome-gut-immune axis which promotes adverse effects of DEHP on the host by altering metabolites sensed by both intestinal and immune T-cells. Wang et al. (2020) postulate that the differences in the toxicity of DEP on different strains and species of rodents are related to the distinctive DEP-induced changes in gut. Lastly, Yang et al. (2019) investigated whether phthalate exposure in newborns through medical treatment that required intravenous infusions had an impact on gut microbiota composition and diversity and found that this type of exposure caused gut dysbiosis, with decreased *Rothia* sp. and *Bifidobacterium longum*. The authors conclude that early-life exposure to DEP alters gut microbiota in newborns and may result in disruptions of immune responses later in life.

### Metals

Many heavy metals have shown endocrine disrupting properties, and human exposure occurs through diet and water, inhalation of polluted air, smoking and dermal absorption (Sabra et al., 2017). The effects of metal exposure on the gut microbiota have been studied in various species including mice, rats, chickens, fish, and humans in adulthood (Table 4).

**TABLE 4:** Studies linking exposure to metals and gut microbiota changes.

**References**	**Animal model**	**Metal dose**	**Microbiota changes**	**Health effects**
**Studies in animals**				
Zhou et al. (2020)	Chickens	Mercuric chloride (250 mg/L)	• At the phylum level, at 30 days there was an increase in the abundance of Proteobacteria and Tenericutes phyla, while Tenericutes phylum increased significantly at 60 days.	• Exposure to mercury reduced body weight. • Possible induction of metabolic disorders (carbohydrate, terpenoids and polyketides, and xenobiotics).
Zhao et al. (2020)	Kunming mice	Mercuric chloride (0 and 80 mg/L)	• Increased abundance *Coprococcus, Oscillospira*, and *Helicobacter*. • Decreased *Lgnatzschineria, Salinicoccus*, and *Bacillus.*	• Increased body weight and glucose levels. • Intestinal injury. • Significantly increased expression of pro-apoptotic genes (Bax, JNK, ASK1, caspase3, and TNF-α), and significantly decreased expression of the anti-apoptotic gene Bcl-2.
Ruan et al. (2019)	Female Kunming mice	Cu and Hg	Decreased abundance of *Rikenella*, *Jeotgailcoccus* and decreased abundance of *Staphylococcus*. *Corynebacterium* increased significantly in mice exposed to Cu. • Decreased abundance of *Sporosarcina, Jeotgailcoccus* and decreased abundance of *Staphylococcus* in the Hg and Cu+Hg groups. *Anaeroplasm* increased significantly in the Cu+Hg group.	• Increased thickness of muscularis internal and externa. • Widened submucosa. • Reduction of goblet cells. • Necrosis of enterocytes. • Decreased height of intestinal villi.
Zheng et al. (2020)	**B. gargarizans tadpoles**	Cu (32 and 64 μg/L), Cr (104 and 416 μg/L), Cd (100 and 200 μg/L), NO_3_ -N (20 and 100 mg/L).	Exposure to NO_3_ -N, Cd, and Cu increased the abundance of *Proteobacteria*. • Exposures to Cu, Cd, Cr, NO_3_ –N increased the abundance of *Bacteroidetes*. • Exposure to Cu and Cd decreased the abundance of *Fusobacteria*.	• Exposure to NO_3_ -N increased the risk of developing metabolic disorders, various diseases, and adaptation to the environment.
Zhang et al. (2020)	**P. clarkii** crayfish	Cd (0, 2, 5, and 10 mg/L)	Firmicutes, Proteobacteria, Bacteroidetes, and Fusobacteria were the predominant phyla in the gut microbiota after exposure to Cd.	• Exposure to Cd could induce histological intestinal damage.
Yan et al. (2020)	**O. melastigma**	Pb (50 μg/L), Cd (10 μg/L), and Zn (100 μg/L)	Increased abundance of Firmicutes *(Lachnoclostridium-10*, Ruminococcaceae and *Lactobacillus*), *Proteobacteria (Burkholderiales and Pseudomonas)*, and *Bacteroidetes* were observed in females treated with Pb, Cd, and Zn. • The gut microbiota of adult males was more sensitive to exposure to Pb, Cd, and Zn than that of females. Exposure in males led to increased abundances of Aurantimonadaceae, *Rhizobium, Rhizobiaceae, Paracoccus, Methylobacterium, Methylobacteriaceae*, and Aurantimonadacea.	• Poor quality of the eggs was observed after exposure.
Richardson et al. (2018)	Sprague-Dawley rats	As (15, 22, or 31 mg/kg), Cd (35, 54, or 85 mg/kg), Co (27, 47, or 82 mg/kg), Cr (44, 62, or 88 mg/kg), Ni (177, 232, or 300 mg/kg)	Exposure to metals resulted in disturbed gut microbiota composition, but the specific taxa affected were not consistent. • The *Proteobacteria* phylum (Enterobactericeae) increased in abundance in response to exposure to high doses of As and Ni.	_
**Studies in humans**				
Shao and Zhu (2020)	Adults	As, Cd, Cu, Pb, Znc (environmental exposure)	Chronic exposure to As, Cd, Cu, Pb, and Zn resulted in disturbances of gut microbiotas in inhabitants of contaminated areas, particularly men. Increased Lachnospiraceae, *Eubacterium elegns*, Ruminococcaceae *UGG-014*, Erysipelotrichaceae *UCG-003, Tyzzerella 3, Bacteroides, Slackia, Italic, and Roseburia*. • Decrease in *Prevotella*.	_
Eggers et al. (2019)	Adults	Pb (environmental exposure)	Increases in microbial α-diversity and richness were associated with higher concentrations of Pb in urine. Proteobacteria abundance increaseds.	_

*Cu, copper; Hg, mercury; Cr, chromium; Cd, cadmium; NO_3_ –N, nitrate ion; Pb, lead; Zn, zinc; As, arsenic; Co, cobalt; Ni, nickel.*

Recent works have described the effects of metals on microbiota including the paper by Zhou et al. (2020) reported that subchronic exposure to mercury in chickens caused dysbiosis and altered microbial growth and development, which may ultimately led to metabolic disorders. Zhao et al. (2020) showed that mercury exposure in mice resulted in alterations in microbial growth and development, metabolic disorders, and promoted apoptosis in mice. Gut dysbiosis was associated with increased abundance of *Coprococcus*, *Oscillospira* and *Helicobacter* and reduced *Lgnatzschineria*, *Salinicoccus*, and *Bacillus*.

Ruan et al. (2019) reported that high exposure to copper and mercury in female mice led to histopathological lesions and changed the diversity of the cecal microbiota. In an amphibian model, Zheng et al. (2020) investigated the effects of exposure to copper, cadmium and chrome on the gut microbiota and found that at the phylum level, copper exposure increased *Proteobacteria*, that has been described to be associated with metabolic diseases (Rizzatti et al., 2017). Copper, cadmium and chrome exposure significantly increased *Bacteroidetes* that is implicated in the protein metabolism and in the complex cycling of carbon (He et al., 2018). Lastly, exposure to copper and cadmium resulted in a reduction of Fusobacteria. The abundance of Fusobacteria is negatively related to the incidence of infectious and diseases producing tissue necrosis (Knutie et al., 2018).

Zhang et al. (2020) found that exposure to cadmium in crustacean *P. clarkii* resulted in histological changes in the intestines and alteration of the richness, diversity, and composition of its gut microbiota with changes in the relative abundances of *Firmicutes, Proteobacteria, Bacteroidetes, Fusobacteria*, and *Actinobacteria*. Yan et al. (2020) investigated the toxicity of environmentally relevant concentrations of metals and microplastics on the gut bacteria and gonadal development in medaka fish and found significant changes in gut microbiota. The authors also reported that the exposure to metals increased the diversity and abundance of intestinal microbiota, in particular of *Proteobacteria*. In an epidemiologic study Shao and Zhu (2020) found that long-term exposure to arsenic, cadmium, copper, lead and zinc increased the relative abundances of *Lachnospiraceae, Eubacterium eligens, Ruminococcaceae UGG-014, Erysipelotrichaceae UCG-003, Tyzzerella 3, Bacteroides, Slackia*, and *Roseburia* and decreased *Prevotella*. The authors also reported that these changes in the microbiota were sex-dependent. Richardson et al. (2018) reported that the microbiota can be used as a pre-clinical marker of exposure to specific heavy metals. They described significant changes in the gut microbial composition in rats after exposure to high doses of chromium and cobalt, and dose-dependent changes after exposure to arsenic, cadmium and nickel.

There are few studies that examine the effects of metal exposure in human microbiota. In this respect, Eggers et al. (2019) examined the association between lead concentration in urine and gut composition in adults and found that increased lead levels were correlated with increased α-diversity and richness. In addition, differences in *Proteobacteria*, including members of *Burkholderiales*, as well as in β-diversity were significantly associated (*p* = 0.003) with differences in lead levels in urine.

### Triclosan, Parabens

Triclosan (TCS) is a well-known EDC added to many consumer products intended to reduce or prevent bacterial contamination (Halden et al., 2017). Parabens are widely used as preservatives in cosmetics, personal care products, drugs, and foods (Quiros-Alcala et al., 2018). Triclosan and parabens have been showed to alter microbiota in different studies (Table 5). Hu et al. (2016) examined the effect of low-dose exposure to DEP, methylparaben (MPB), TCS and the effect of simultaneous exposure (MIX) in the gut bacterial composition in adolescent rats and found an increased relative abundance of *Bacteroidetes* (*Prevotella*) and reduced relative abundance of *Firmicutes* (*Bacilli*) in exposed rats versus controls. The authors also found an increased abundance of *Elusimicrobia* in rats exposed simultaneously to DEP and MPB, increased *Betaproteobacteria* in simultaneous exposure to MPB and MIX, and *Deltaproteobacteria* in TCS-exposed rats. Surprisingly, these differences in gut bacterial composition decreased diminished by adulthood despite continued exposure, suggesting that environmental exposure to these chemicals during adolescence has a strongest effect on the gut microbiome. They also observed that exposure during adolescence, especially to DEP and MPB, led to a slight but consistent reduction in the bodyweight, consistent with the reduced *Firmicutes/Bacteroidetes* ratio found in exposed adolescent rats. It has been reported that the gut microbiota of obese animals and humans shows a higher *Firmicutes*/*Bacteroidetes* ratio compared with normal-weight individuals, suggesting this ratio as a biomarker. However, the actual evidence of this association is not convincing (Magne et al., 2020).

**TABLE 5:** Studies linking exposure to triclosan and parabens and microbiome changes.

**References**	**Animal model**	**Triclosan and Paraben dose**	**Microbiota changes**	**Health effects**
**Studies in animals**				
Gaulke et al. (2016)	Zebrafish	TCS (100 μg/g)	• Exposure to TCS led to a slight reduction in diversity, with significant decreases in α-diversity between days 4 and 7 in exposed fish. • Reduced abundance of *Enterobacteriaceae* and increased abundance of *Pseudomonas*.	_
Narrowe et al. (2015)	Fathead minnow	TCS (100 and 1,000 ng/L)	• Increase in α-diversity associated with TCS exposure.	• Exposure to TCS may induce long-term effects on the host organism.
Hu et al. (2016)	Sprague-Dawley rats	MPB (0.105 mg/kg) and TCS (0.05 mg/kg)	• The abundance of *Bacteroidetes* (*Prevotella*) and *Elusimicrobia* increases in both groups. • Firmicutes (*Bacilli*) abundance decreased in both groups. • Betaproteobacteria abundance increased in the group exposed to MPB. • Deltaproteobacteria increased in the group exposed to TCS.	• A subtle but constant reduction in body weight was observed in the young rats.
Yang et al. (2018)	C57BL/6 male mice	TCS (5–80 mg/kg)	• Beneficial bacteria like *Bifidobacterium* were reduced.	• Exposure to TCS led to low-grade colon inflammation and colitis and increased risk of colon cancer related to the presence of colitis.
Gao et al. (2017)	C57BL/6 mice	TCS (2 mg/L)	• After 4 weeks of exposure, α-diversity increased but it subsequently decreased after 9 weeks of exposure.	• The bacterial genes involved in stress response showed significant enrichment after exposure to TCS. • High enrichment of metal resistome in the gut microbiota, which is related to a reduced effectiveness of infection treatment.
**Studies in humans**				
Bever et al. (2018)	Infants	TCS (exposure through breast milk)	• Significant increases in the genus *Dermabacter*, order Rhodospirillales, and family Rhodospirillaceae were found in babies exposed to TCS through breast milk.	_
Ribado et al. (2017)	Infants and their mothers	TCS (environmental exposure and through breast milk)	• Enrichment of the phylum *Proteobacteria* in mothers exposed to TCS-containing toothpaste. • Enrichment of *Proteobacteria* in infants with higher TCS levels.	_

*TCS, triclosan; MPB, methylparaben.*

In a zebrafish model Gaulke et al. (2016) found that TCS exposure resulted in alterations in the composition and ecological dynamics of gut microbial communities. TCS exposure was also related to rapid changes in microbiome structure and diversity, with disturbance of *Enterobacteriaceae*. TCS-induced changes in gut microbiota have also been found in other animal models, including fathead minnows (Narrowe et al., 2015), rats (Hu et al., 2016), and mice (Gao et al., 2017; Yang et al., 2018).

In contrast, only few studies have investigated the effects of TCS exposure on the gut microbiota in humans. Bever et al. (2018) found that fecal microbiota diversity in infants exposed to triclosan through to breast milk was different from that found in infant fed milk with non-detectable levels of TCS. In addition, higher relative abundance of genus *Dermabacter* was observed in children exposed to breast milk containing TCS. Ribado et al. (2017) reported that chronic exposure to TCS through toothpaste increased the relative abundance of broadly antibiotic-resistant *Proteobacteria* species in adults and in children with high TCS levels in urine.

### Polybrominated Diphenyl Ethers (PBDEs)

PBDEs are environmentally persistent chemicals widely used as flame retardants (Poston and Saha, 2019) that have been shown to alter microbiota (Table 6). In a zebrafish model, Chen et al. (2018b) showed that exposure to environmentally realistic concentrations of PBDEs resulted in alterations in the gut microbial community in a sex-dependent manner, significantly affecting zebrafish health. Wang et al. (2018) found that perinatal exposure to bromodiphenyl ether (BDE-47) in an obese rat model led to obesity, hepatic steatosis, and dysfunctional glucose homeostasis and metabolism. The authors also reported that exposure to BDE-47 led to changes in gut microbiota diversity and composition, and microbial metabolism. These exposure-related changes were stronger in mice on a high-fat diet. These findings suggest that early life exposure to BDE-47 at low doses of may stimulate obesity and the development of metabolic dysfunction.

**TABLE 6:** Studies linking exposure to polybrominated diphenyl ethers and microbiota changes.

**References**	**Animal model**	**PBDE dose**	**Microbiota changes**	**Health effects**
**Studies in animals**				
Chen et al. (2018b)	Zebrafish	PBDE mixture (DE-71) (5 ng/L)	• Higher relative abundance of Firmicutes and Bacteroidetes in the gut of male fish, but a lower Firmicutes/Bacteroidetes ratio was observed. • DE-71 led to decreased Bacteroidetes in the gut of female fish with a higher Firmicutes/Bacteroidetes ratio. • *Mycoplasma, Ruminiclostridium*, *unclassified* Firmicutes *sensu stricto* and *Fusobacterium* were not detected in the gut of male and female fish.	• In males, an alteration in intestinal health was observed due to exposure to DE-71, which led to disruptions of the neural signaling, of the integrity of the epithelial barrier, inflammatory response, oxidative stress and antioxidant capacity, as well as disruptions of the detoxifying capacity. • In females, the physiological activities of the intestine remained unchanged.
Wang et al. (2018)	Mice	PBDE-47 (0, 0.002 and 0.2 mg/kg)	• Exposure resulted in decreased abundance of *Bacteroidetes* and *Proteobacteria* and in an increase of *Actinobacteria* at the phylum level. • Exposure resulted in increased abundance of *Candidatus_Saccharimonas, Ruminococcaceae_UCG-013, Staphylococcus, Gemella, Eubacterium_nodatum_group, Corynebacterium_1* and *Paenalcaligenesen* and in a decrease in the abundance of *Turicibacter* and *Anaerotruncus* at the genus level.	• High fat diet-induced obesity increased as a result of the exposure to BDE-47. • Steatosis of the liver, disturbances in glucose homeostasis, metabolic dysfunction, and altered levels of gene mRNAs involved in lipid metabolism were found in mice fed high-fat diet and exposed BDE-47.
Li et al. (2017)	Male C57BL/6 mice	PBDE-47 (10–100 μmol/kg) and PBDE-99 (10–100 μmol/kg)	_	• Absence of gut microbiome increased PBDE-99-mediated upregulation of many genes involved in drug metabolism and it also affected hydroxylation of PBDEs. • Exposure to PBDE increased unconjugated bile acids in multiple bio-compartments in a gut microbiota-dependent manner.
**Studies in humans**				
Laue et al. (2019)	Children	PBDE-47, PBDE-99, PBDE-100, PBDE-153 (environmental exposure)	• Exposure to PBDE-99 was associated with a decrease in uncultured bacteria within the Ruminococcaceae *NK4A214* group and exposure to PBDE-47 led to differences in *Ruminococcus 2*.	_

*PBDEs: Polybrominated diphenyl ethers; DE-71: PBDE mixture; PBDE-47: 2, 2′, 4, 4′-tetrabromodiphenil ether; PBDE-99: 2, 2′, 4, 4′, 5-pentabromodiphenil ether; PBDE-100: 2,2′,4,4′,6-pentabromodiphenyl ether; PBDE-153: 2,2′,4,4′,5,5′-Hexabromodiphenyl ether.*

Li et al. (2017) investigated whether exposure to PBDEs is associated with dysbiosis in standard and germ-free mice and reported that PBDE exposure resulted in a significant decrease in the alpha diversity of gut microbiome and modulated 45 bacterial species with increased *Akkermansia muciniphila* and Erysipelotrichaceae *Allobaculum* spp., which have shown antiinflammatory and antiobesity capacity. Lastly, they also found that PBDE exposure increased the amount of unconjugated bile acids in a gut microbiota-dependent manner.

Laue et al. (2019) compared the effects of PBDE perinatal exposure on the gut microbiome profiles with the effects of exposure in mid-childhood. Higher PBDE concentrations were related to lower abundances of uncultured bacteria in the *Ruminococcaceae NK4A214* group and with variable abundances of *Ruminococcus 2* species. These changes at the taxon-level did not lead to differences in within- or between-subject diversity. Lastly, exposures at delivery were not linked to differences in taxa.

## Conclusion

Endogenous steroid hormones and EDC interact with gut microbiota through different pathways: (i) they could be directly metabolized by the gut microbiota after ingestion or after being conjugated in the liver, and (ii) they can interfere with the composition and/or metabolic activity of the gut microbiota. Both interactions may have an effect in host health. We propose that toxicological studies should consider the changes in the gut microbiota related to contaminant exposure and term the chemicals having an effect on gut microbiota as “microbiota disrupting chemicals.” The risk of developing certain disorders associated with gut microbiota changes should be established by determining both the effects of the MDC on gut microbiota and the impact of microbiota changes on chemicals metabolism and host susceptibility.

Gut microbiota composition used in combination with the determination of serum, urinary and fecal levels of estrogens can be used as susceptibility or risk biomarkers for disease. Profiling of the gut microbiome could be taken a step further by performing metagenomic analysis to determine the levels of genes encoding β-glucuronidases and other enzymes involved in hormone metabolism in the context of hormone-related diseases. Novel hormone modulation methods involving modifications of the microbiome or control of hormone and EDC exposure may be very effective and provide an alternative to current treatments of hormone-dependent disorders. Additionally, the therapeutic effects of bacteria on the endobolome, which cluster biosynthetic and metabolic arsenal for estrogens, steroid hormones and/or endocrine disruptor chemicals, should also be considered for the design and development of biotherapeutic products. The endobolome plays a central role in the gut microbiota as seen by the amount of potentially endobolome-mediated diseases and thereby it can be considered an attractive diagnostic tool and therapeutic target for future research strategies that envisage the use of next generation of probiotics.

In any case, further animal controlled experiments, clinical trials and large epidemiological studies are required in order to establish the concatenated impact of the microbial disrupting chemical-microbiota-host health axis.

## Author Contributions

MA and AR: conceptualization. MA, AR, and YG-O: methodology, writing – original draft preparation, review, and editing. All authors have read and agreed to the published version of the manuscript.

## Conflict of Interest

The authors declare that the research was conducted in the absence of any commercial or financial relationships that could be construed as a potential conflict of interest.
